# Emerging New Biomarkers for Cardiovascular Disease

**DOI:** 10.3390/ijms23063274

**Published:** 2022-03-18

**Authors:** Luc Rochette

**Affiliations:** Physiopathologie et Epidémiologie Cérébro-Cardiovasculaires (PEC2), Faculté des Sciences de Santé, Université de Bourgogne—Franche Comté, 7 Bd Jeanne d’Arc, 21000 Dijon, France; luc.rochette@u-bourgogne.fr

In this Special Issue of the *International Journal of Molecular Sciences*, we include insightful reviews and research papers on the subject “Emerging New Biomarkers for Cardiovascular Disease”. We would firstly like to share some background about this topic concerning the concept of the “biomarker”. Innovative and specific biomarkers can serve as new diagnostic markers for the detection of cardiovascular disorders to guide the prognostics and emerging therapeutics.

A biomarker is defined as “a characteristic that is objectively measured and evaluated as an indicator of normal biological processes, pathogenic processes, or pharmacologic responses to a therapeutic intervention” [1]. Biomarkers are, by definition, quantifiable characteristics of biological processes, but to identify biomarkers requires the determination of their relevance and validity [2]. Inflammation-related markers are prominent among the most validated biomarkers that are currently in use. Innovative biomarkers have emerged as relevant contributors in the energy homeostasis field and have appeared as valid biomarkers of various cardiovascular and metabolic diseases. Among these presumed and specific biomarkers, several members of the TGF-Beta super-family, GDF15, GDF11, newly emerging cardiokines, miRNAs, and markers discovered via proteomics in relation to oxidative stress are all involved in cardiovascular disease. The evaluation of their circulating levels might provide new insights into the course of disease [3,4,5].

This Special Issue is composed of three articles and four reviews that are briefly outlined below. Pozo-Agundo and coauthors [6] report for the first time the specific signature of differentially expressed miRNAs transported in plasma exosomes associated with hereditary hemorrhagic telangiectasia (HHT). Exosomes released from cells have been shown to carry different nucleic acids, including microRNAs (miRNAs). miRNAs significantly regulate cell growth and metabolism by the posttranscriptional inhibition of gene expression [7,8]. HHT is a vascular dysplasia with autosomal dominant traits characterized by recurrent and spontaneous nose bleeds (epistaxis), mucocutaneous diseases, and arteriovenous malformations (AVM) in the internal organs: lungs, liver, and brain. The bioinformatics analysis points out the biological functions affected in HHT. The majority of them have strong diagnostic value and allow us to discriminate between HHT1 and HHT2. The data indicate that the cellular components of arteriovenous malformations determine this exosomal miRNA signature. The results suggest a key functional role of these exosomal miRNAs in the pathophysiology of HHT. HHT is a rare disease with an estimated prevalence of around 1/8000. HHT patients may appear to have no symptoms until their epistaxis is strong, in around the third or fourth decade of life. This situation usually causes a delay in diagnosis of many years, which prompts the need for early molecular diagnosis. The results of this study provide stimulating possibilities for molecular diagnosis and might help to establish disease-specific biomarker signatures.

O’Toole and coauthors [9] and Oosterwijk and coauthors [10] conducted complementary studies designed to specify the role of carnosine and calprotectin in patients with cardiovascular diseases (CVDs) and metabolic disorders. Carnosine belongs to the family of histidyl dipeptide. It is a naturally occurring dipeptide (β-alanine-L-histidine) found in abundance in highly metabolic tissues such as skeletal muscle, heart, and brain. These peptides can bind to endogenous aldehydes, such as 4-hydroxy trans-2-nonenal (HNE) or acrolein, which are produced by oxidative stress. Carnosine can quench singlet oxygen and chelate metals, with these properties being implicated with a protective role in several diseases [11]. The results of the clinical and biochemical studies proposed in the Special Issue demonstrated significant associations between the levels of carnosine, the conjugates carnosine-propanal and carnosine-propanol, and the presence of CVD risk factors, suggesting that carnosine and its conjugates may be novel biomarkers of CVD risk and adverse outcomes. This study is the first analysis assessing the association of nonconjugated carnosine, carnosine-propanal, and carnosine-propanol with indices of CVD risk or overt CVD. Calprotectin, also known as S100A8/S100A9 complex, is a protein that is secreted by leukocytes and its expression is upregulated in response to inflammation [12]. Elevated serum concentrations of calprotectin have been reported in multiple chronic inflammatory diseases, including insulin resistance and obesity. Circulating calprotectin is a potential biomarker for endovascular inflammation in type 2 diabetes mellitus (T2DM). Oosterwijk and coauthors [10] investigated the determinants of calprotectin and its relationship with the presence of CVD in 362 T2DM patients included in the Diabetes and Lifestyle Cohort Twente-1 (DIALECT-1) study. Plasma calprotectin was found to be independently associated with smoking status, intake of mono- and disaccharides, and albuminuria. Higher serum calprotectin levels were associated with smoking, a cause of endovascular inflammation. Elevated levels of inflammatory markers may be the result of pre-existing atherosclerosis in subjects with albuminuria. Moreover, plasma calprotectin level is associated with the presence of CVD in patients with T2DM. In these conditions, the prognostic value of calprotectin in patients with T2DM may have several implications for the training of strategies to prevent chronic vascular complications.

This Special Issue aims to delineate the pathophysiology of myocardial disease and new biomarkers. Novel cardiovascular biomarkers provide mechanisms by which to more accurately risk stratify patients and can aid in the diagnosis and prognosis of patients. Janjusevic and coauthors [13] reported the current knowledge of the biomarkers used in clinical practice and explored new markers, such as non-coding RNA and exosomes, which may help with heart failure (HF) diagnosis in the asymptomatic phase. The aim of the review is to collect the current knowledge on past and novel biomarkers in the early diagnosis of cardiac dysfunction in asymptomatic individuals. A number of studies have investigated the role of Long Non-Coding RNAs (lncRNAs) in cardiac physiology and pathology, identifying a set of new candidate biomarkers. Therefore, their pathological role in HF remains to be confirmed. Further clinical studies are required to evaluate the prognostic power of these non-coding RNAs in the development of cardiac deterioration at its initial stage of HF. Concerning the exosomes as biomarkers, aside from the promising results that suggest exosome suitability for diagnostic purposes, several limitations hamper their use in clinical practice. The understanding of invasive and noninvasive specific biomarkers may yield valuable insights into the pathophysiology and prevention of CVD [4]. Studies towards plasma biomarkers in genetic cardiomyopathy have been hindered for a long time because of the absence of large cohorts and the time has come to study significant cohorts. A great number of pathogenic gene variants have been identified and this number is likely to increase; some genes can be linked to a specific cardiomyopathy. In the review: “The time has come to explore plasma biomarkers in genetic cardiomyopathies”, Stege and coauthors [14] discuss the potential use of conventional plasma biomarkers, including natriuretic peptides and troponins, and the use of novel biomarkers, such as cardiac autoantibodies in genetic cardiomyopathy, in cardiomyopathy cohorts. It is essential to underline that cardiomyopathies are not defined by a specific genetic mutation, but by definite morphological and functional cardiac alterations. Selecting the optimal time for treatment is the challenge in the relationship with specific biomarkers.

HF is an end-stage cardiovascular syndrome with high morbidity and mortality. The morbidity is caused by impaired contractility and relaxation-induced pump failure, as well as sudden cardiac death [15]. Despite promising results in animal models, clinical trials of gene therapy for HF have not yet shown positive results. Cardiomyocyte transverse tubule (t-tubule) microdomains organized by the membrane scaffolding protein cardiac-bridging integrator 1 (cBIN1) appears as an important regulator of beat-to-beat myocardial contraction and relaxation. In the Special Issue, Li and coauthors [16] conducted a review on the cardiac t-tubule cBIN1-microdomain, a diagnostic marker and therapeutic target of HF. The authors reported their own studies that indicate that the pathophysiology associated with cBIN1-microdomain remodeling in failing hearts can be normalized by exogenous cBIN1 introduced by gene therapy. In this review, the authors discuss the mechanisms underlying the blood availability of cBIN1 and recent clinical studies of cBIN1 that evaluate its clinical usage as a biomarker, helping HF diagnosis and prognosis. It is well documented that oxidative stress (OS) plays an important role in biology and induces vascular-related gene expression, promoting local inflammatory response and cardiovascular dysregulation. When OS occurs, vascular walls produce excessive reactive oxygen species (ROS), which cause damage to the structure and function of endothelial cells [17,18]. Among the endogenous compounds, thioredoxin-interacting protein (TXNIP) is a metabolism oxidative- and inflammation-related marker induced in cardiovascular pathologies. Domingues and coauthors [19] reviewed the recent advances in the functions of TXNIP as a marker of cardiovascular risk and diseases. Thioredoxin (TRX), a major antioxidant enzyme, is carried by TXNIP to the membrane, and this interaction is fundamental. Several studies suggest that TXNIP may bind to the NLRP3 inflammasome, which enhances the inflammatory response. TXNIP has also attracted attention due to its wide-ranging functions impacting several aspects of energy metabolism; TXNIP modulates cellular glucose utilization, the cellular redox status, and the mitochondrial oxidation of metabolic substrates. It has been reported that the interaction between TXNIP and NLRP3 is involved in damage-induced ischemia. The balance of the TRX-TXNIP system is essential for the survival of cells in the context of myocardial or cerebral ischemia. Finally, the plasma levels of TXNIP may serve as a potential biomarker in cardiovascular and ischemic diseases, but also as a novel identified target for preventive and curative medicine.

Taking into account the multi-disciplinary character of this Special Issue, we hope that it will provide innovative information to researchers and clinicians to guide the prognostic and emerging therapeutics.

## Data Availability

Not applicable.
